# Can cardiac MRI be the index metric for risk stratification in dilated cardiomyopathy; the impact of an LV mid-myocardial stripe on subsequent risk of LVAD, transplantation and death

**DOI:** 10.1186/1532-429X-14-S1-P147

**Published:** 2012-02-01

**Authors:** Jose Venero, Mark Doyle, Srinivas Murali, Diane A Vido, Vikas K Rathi, Saundra Grant, June Yamrozik, Ronald B Williams, Raymond Benza, George Sokos, Peter Olson, Robert W Biederman

**Affiliations:** 1 Allegheny General Hospital, Pittsburgh, USA

## Summary

The mid-wall intramyocardial 'stripe' is now shown to be robust in its prediction of subsequent need for LVAD and/or transplantion and death out to 12 months following index CMR exam.

## Background

Patients with newly diagnosed dilated cardiomyopathy (DCM) and advanced heart failure have a very high morbidity and mortality with an unpredictable clinical course. We investigated the role of CMR via LGE (late gadolinium enhancement) in this cohort of high-risk patients.

Hypothesis Utilizing cardiovascular MRI (CMR), we assessed the prognostic value of LGE in primary dilated DCM patients referred for possible transplantation/LVAD consideration.

## Methods

Over 49 consecutive months, 61 consecutive DCM pts were referred for standard 3D CMR (1.5T,GE) to interrogate the LV pattern, distribution and extent of LGE (MultiHance, Princeton, NJ). Inclusion criteria for a primary non-ischemic dilated DCM and EF <45% were met in 31 pts. DCM were categorized into: 1) +midwall LV Stripe 2) −midwall LV Stripe groups. MACE defined by the composite of death, need for LV assist device (LVAD), and urgent cardiac transplantation (Tx) were evaluated over the ensuing 12 months.

## Results

There were no differences between groups for demographics, baseline LVEF, NYHA class or invasive hemodynamics. There were 18/31 (58%) pts with + mid-wall Stripe. Nine events occured: 7 pts required urgent Tx ± LVAD and 2 pts died among the 31 pts. The group with +Stripe categorization strongly predicted the need for LVAD, urgent Tx surgery and death over the ensuing 12 months (Kaplan-Meier: X2= 9, p<0.005). Specifically, all the events occured in the +Stripe pts: 7/18 (39%) +Stripe pts required urgent Tx or LVAD by 6 months and 2/18 (11%) +Stripe pts died by 12 months. The −Stripe group never experienced the need for Tx, LVAD or death, indeed revealing signs of improvement in NYHA and LVEF (p<0.05). Neither LVEF, LVEDV, RVEF, RV size, PCWP, PAP, NYHA or age predicted MACE via univariate analysis. LVEDD was statistically different between +Stripe and −Stripe group but by Multivariate analysis did not predict MACE.

## Conclusions

The presence of +Stripe on CMR is strongly predictive of LVAD, transplant need and death over the ensuing 12 months in DCM patients. All −Stripe pts possess a 100% favorable sign through one year. Incorporating CMR imaging into routine clinical practice may help to identify DCM risk; conservatively manage low-risk patients while expectantly manage high-risk patients.

## Funding

Internal.

